# New York Letter

**Published:** 1892-06

**Authors:** Wm. L. Russell

**Affiliations:** 151 East 50th Street; New York


### *CORRESPONDENCE.*

---

NEW YORK, May 16, 1892.

### MIDDLETON GOLDSMITH LECTURE.

This lecture is under the auspices of the Pathological Society, and is the outcome of an annual prize provided for by one of the founders of the Society. The lecturer this year was Dr. F. P. Kinnicutt, who chose for his subject, "The Present Status of the Treatment of Tuberculosis," special reference being made to the more recent contributions. The part played by the bacillus in the production of tuberculosis was assumed as a settled fact, and the changes produced within the lungs received a passing notice. He stated that the bacillus was the exciting factor in the production of these lesions which consisted in a diffused or aggregated epithelial cell growth, coagulation necrosis, excavation, inflammatory consolidation, bronchitis and peribronchitis. The therapeutic measures suggested were prophylactic and remedial, the former having in view the destruction of the bacillus without the body, the increase of the resistive power of the system to its attack, and the mimimizing of its effects after its introduction had been affected. The latter had for their object the destruction of the bacillus within the body, the modification of its action, and the increase of the germicidal power of the tissues. It had been found that the bacilli were not in the air expired by the tuberculosis patient, but were in the sputum in large numbers. It was known, too that they were incapable of escaping from a fluid medium and, it was only when the sputum became dried that they were disseminated through the air. The stools contained bacilli, and also the milk and meat of diseased animals. The examination of the dust of hospital wards showed the possibility of inducing tuberculosis in animals by inoculation with that from the medical wards. That from the surgical gave negative results. Dust from the rooms of patients in private homes showed an ability to produce the disease, although when spittoons were used, the results were negative.

The contagiousness of tuberculosis had been investigated by several, and many striking instances had been noted. It was shown that during the past twenty-five years, eighty-eight per cent. of the deaths among the Prussian nursing sisters was due to this disease. In nearly half the convents the percentage was seventy-five and in two tuberculosis was the sole cause of death. The death rate in these convents rose rapidly on the introduction of a case of phthisis. The production of tuberculosis by the milk of diseased cows had been demonstrated in our own country. As illustrations of direct contagion, a case had been reported in which the ear-rings of a victim of the disease, had produced a tubercular adenitis, and eventually systemic infection in a healthy women by whom they were worn ; another in which the vesicles of an eczematous child who slept with a consumptive mother, showed the presence of the bacillus after a year of exposure ; in still another the saliva of an operator who was accustomed to perform the Jewish right of circumcision infected several children.

The vital importance of disinfecting or destroying sputum was apparent. Chemical disinfectants were, as a rule, ineffective, the bacilli remaining alive in a ten per cent. carbolic solution for twenty-four hours. Cleansing with boiling water was not sufficient for sputum cups, and was not without danger to those who emptied and cleansed them, infection through abrasions on the hands having been known to occur. Sterilization by steam was an effective method of cleansing these cups. A still better plan, however, was the use of small paper boxes. They were cheap and convenient, and could be burnt every day. The use of handkerchiefs, and of the floor, as depositories for sputum ought to be distinctly discouraged. The walls and floors of patients rooms should be cleansed by wiping with damp cloths, not by sweeping. The protection of our milk supply demanded legislation for securing a proper inspection of dairies and slaughter-houses. Such measures as these were more likely to prove effective than those intended to increase the resistive power of the individual, and if the scourge were to be eliminated, it must be through their adoption.

Among remedial measures none was more important than that introduced by Koch, not so much from what it had directly accomplished, as from the direction in which it pointed and the field which it had opened. Further investigations of tuberculin showed it to be composed of at least three substances, one an albuminose, a second an alkaloid, and the third an extractive. The familiar reaction was caused by the proteid. This had now been isolated, and had been named tuberculositin. Experiments had been made with it abroad, and in St. Luke's Hospital in this city. It had germicidal power, and was capable of destroying the bacilli in the body without producing inflammation. In bad cases the removal of the first cause did not cure. Some of the constituents of tuberculin were harmful. It seemed highly probable that continued experiments with it, however, and with its constituents would result in a modification of it, or in the discovery of a new substance that would prove to be the remedial agent wished for.

The power of cantharides to produce a serous exudation when taken internally had suggested its use in tuberculosis, as the serum was known to have germicidal power, and to stimulate cell action and nutrition. The cantharidates of sodium and potassium had been tried, but the remedy had taken no permanent place.

Injections of the serum of dog's blood was said to prevent the development of experimental tuberculosis, half a cubic centimeter to each kilometer of weight of the rabbit being the amount employed.

Chloride of zinc had also been suggested, as it was said to form a combination with the albuminoids of the blood which was germicidal. This was questionable, and in any case man was not a test tube. Good results were said to have been obtained however in cases of tuberculosis of joints and glands, the morbid cells being destroyed, and sclerotic tissue being produced. Two drops of a ten per cent. solution were injected in a number of places around the diseased area.

Creasote was introduced as a remedy in 1830, but fell into disuse very soon, and was reintroduced in 1887. The evidence in current literature seemed to be favorable to its value. It arrested fermentative processes in the digestive tract, and stimulated the appetite, nutrition being improved in consequence. Some had thought that as a solution of one to four thousand devitalized the bacilli in a test tube, it was possible to charge the blood to this extent and thus destroy the bacilli in the tissues. It entered into combination with albuminoids of the blood however, and ceased to be germicidal. The general opinion of observers was that creasote acted by improving nutrition and not from any specific influence.

HYDROTHERAPY.

The strongest advocate and most scientific exponent of this valuable addition to our therapeutic resources in this city is Dr. Simon Baruch. During a recent discussion at the Academy he made a further plea for its more general and careful employment. He said that he regarded the external application of water as one of the most potent remedies in our possession. Its action was explicable on a rational basis, and the dosage could be accurately adapted to the condition. Its effect was produced chiefly through the nervous system. The simple illustration seen in the effect of a dash of water in the face of a fainting person showed what a powerful excitant of reflex action it was. The mechanical and thermic action of the water thus could be controlled accurately by means of the pressure gauge, the thermometer and the clock, and it thus became a most scientific and controllable agent. The technique was thought to be difficult, but this was not so if the rationale of its action were once passed. It had not become popular because of inconvenience, and prejudice of the people.

Dr. M. A. Starr said that he had found baths and douches of great value in the treatment of nervous diseases. Neurasthenic cases were best treated in institutions. Organic diseases of the cord, as locomotor ataxia, and lateral and multiple sclerosis were all benefited by the application of hot and cold water to the back by means of a sponge or a douche. Obscure nervous diseases in delicate women might be treated by the wet sheet, with rubbing, or by the douche, cold and hot alternating.

### REACTION OF ETHER WITH URINE.

Dr. Andrew H. Smith has been engaged for several years in a series of experiments showing the reaction of ether with urine. He gave an account of his work and a demonstration of his results at the last meeting of the Academy. He stated that he had been led to undertake the experiments through the statement which had been made that egg albumen was soluble in ether while serum albumen was not. He had thought that the amount of alimentary substances which escaped by the urine might perhaps be determined by this fact. He had been surprised however at the reaction which he had observed in healthy urine. When great quantities of ether and urine were well shaken together in a test tube and then allowed to stand, a gelatinous opalescent stratum was observed to collect on the surface. So tenacious was this that the test tube could be inverted without escape of its contents. If this coagulum were removed by filtration, a further supply could be obtained by treating the filtrate with acetic acid, and more ether. He had found that urine could be divided into three classes, in one of which no reaction could be obtained, in a second it could be obtained with ether alone, and in the third with ether and acid combined. The coagulum was not due to mucin as it was obtained after all of this substance had been removed. In young children and in dyspeptics little deposit could be obtained. The quantity seemed to be proportionate to the appetite and vigor of digestion. It increased when an individual went to the seaside. In Bright's disease it was very dense, where albumen was present, and was still found, although in smaller quantity, when the albumen had been removed. The presence of sugar did not modify the reaction.

The significance and value of the experiments could not yet be determined. It was certain however that during the process of elimination of ether by the kidneys after anæsthesia it was likely to come in contact with the urine in the tubules and such circumstances as would render probable the deposit of this tenacious gelatinous substance. It was possible that some cases of suppression were due to this cause. The advantage of a low diet, and of the production of an alkaline reaction of the urine before ether anæsthesia thus became a matter of much importance.

WM. L. RUSSELL.

151 *East 50th Street.*

